# Bioinformatic analysis of underlying mechanisms of Kawasaki disease via Weighted Gene Correlation Network Analysis (WGCNA) and the Least Absolute Shrinkage and Selection Operator method (LASSO) regression model

**DOI:** 10.1186/s12887-023-03896-4

**Published:** 2023-02-24

**Authors:** Yaxue Xie, Hongshuo Shi, Bo Han

**Affiliations:** 1 Department of Pediatrics, Shandong Provincial Hospital, Shandong University, Jinan, 250021 Shandong China; 2 College of Traditional Chinese Medicine, Shandong University of Traditional Chinese Medicine, Jinan, 250021 Shandong China; 3 Department of Pediatrics, Shandong Provincial Hospital Affiliated to Shandong First Medical University, Jinan, 250021 Shandong China

**Keywords:** Kawasaki disease, Weighted gene correlation network analysis, LASSO regression model, ceRNA network, CIBERSORT

## Abstract

**Background:**

Kawasaki disease (KD) is a febrile systemic vasculitis involvingchildren younger than five years old. However, the specific biomarkers and precise mechanisms of this disease are not fully understood, which can delay the best treatment time, hence, this study aimed to detect the potential biomarkers and pathophysiological process of KD through bioinformatic analysis.

**Methods:**

The Gene Expression Omnibus database (GEO) was the source of the RNA sequencing data from KD patients. Differential expressed genes (DEGs) were screened between KD patients and healthy controls (HCs) with the “*limma*” R package. Weighted gene correlation network analysis (WGCNA) was performed to discover the most corresponding module and hub genes of KD. The node genes were obtained by the combination of the least absolute shrinkage and selection operator (LASSO) regression model with the top 5 genes from five algorithms in CytoHubba, which were further validated with the receiver operating characteristic curve (ROC curve). CIBERSORTx was employed to discover the constitution of immune cells in KDs and HCs. Functional enrichment analysis was performed to understand the biological implications of the modular genes. Finally, competing endogenous RNAs (ceRNA) networks of node genes were predicted using online databases.

**Results:**

A total of 267 DEGs were analyzed between 153 KD patients and 92 HCs in the training set, spanning two modules according to WGCNA. The turquoise module was identified as the hub module, which was mainly enriched in cell activation involved in immune response, myeloid leukocyte activation, myeloid leukocyte mediated immunity, secretion and leukocyte mediated immunity biological processes; included type II diabetes mellitus, nicotinate and nicotinamide metabolism, O-glycan biosynthesis, glycerolipid and glutathione metabolism pathways. The node genes included ADM, ALPL, HK3, MMP9 and S100A12, and there was good performance in the validation studies. Immune cell infiltration analysis revealed that gamma delta T cells, monocytes, M0 macrophage, activated dendritic cells, activated mast cells and neutrophils were elevated in KD patients. Regarding the ceRNA networks, three intact networks were constructed: NEAT1/NORAD/XIST-hsa-miR-524-5p-ADM, NEAT1/NORAD/XIST-hsa-miR-204-5p-ALPL, NEAT1/NORAD/XIST-hsa-miR-524-5p/hsa-miR-204-5p-MMP9.

**Conclusion:**

To conclude, the five-gene signature and three ceRNA networks constructed in our study are of great value in the early diagnosis of KD and might help to elucidate our understanding of KD at the RNA regulatory level.

**Supplementary Information:**

The online version contains supplementary material available at 10.1186/s12887-023-03896-4.

## Introduction

Kawasaki disease (KD), termed mucocutaneous lymph node syndrome, is a kind of febrile systematic vasculitis of unknown etiology, that affects children under 5 years old. The pathophysiological mechanisms of KD involve the ectopic activation of the immune system and overwhelming release of proinflammatory cytokines which can result in abnormalities in micro and medium vessels, especially coronary arteries (CA) [1, 2]. The standard regimen of intravenous immunoglobulin (IVIG) at 2 g/kg with aspirin intervention between Days 5–7 of the illness can significantly decrease the incidence of CA lesion (CAL), which is the most frequent ensuing catastrophic issue [3, 4], and could progress into CA aneurysm (CAA), stenosis, thrombosis, and myocardial infarction [2]. Unfortunately, delayed treatment and resistance to IVIG of 10%-20% of patients give rise to the development of CAA. The Current diagnosis of KD is mainly based on a collection of clinical features, including fever (usually ≥ 39 °C) for more than 5 days, strawberry tongue, cracked lips, bilateral conjunctivitis, cervical lymphadenopathy, edema of the extremities, general rash, and CAL detected on echocardiography. The adjunctive diagnoses mostly engage inflammation biomarkers, such as erythrocyte sedimentation rate, white blood cell/leukocyte count, platelet count, C-reactive protein, interleukin-6 (IL-6), and serum albumin levels [5, 6].

Bioinformatic analysis of microarray data is one of the first well-established methodologies for the high-throughput analysis of biological systems. With the advances in computer technology, many gene expression profiling studies of KD have been performed to discover its pathogenesis. Weighted Gene Coexpression Network Analysis (WGCNA) is an unsupervised clustering approach that focuses on the gross expression profile of genes to avoid gene loss, thus providing a comprehensive description of cellular responses and aiming to discover the associations between genes, modules and phenotypes [7, 8]. The LASSO regression model is a multigene-based classifier, which is a prevailing high-dimensional variable regression analysis algorithm [9]. The combination of WGCNA and LASSO represents a credible approach to reveal novel biomarkers. Additionally, the construction of competitive endogenous RNA (ceRNA) networks will help to illustrate the novel mechanisms of transcriptional regulation.

To date, articles about KD have mainly focused on the following aspects. First, it is difficult to differentiate KD from other febrile diseases, such as bacterial and viral infections and systemic lupus erythematosus (SLE), because of the atypical clinical symptoms and laboratory indicators. Previous papers have ascertained optimal signatures at different levels, including whole blood, specific cell type, urine peptidome and clinical information, to sequester KD from clinically confusing diseases to prompt timely treatment and to prevent the occurrence of complications [10–13]. Second, KD patients who are unresponsive to the administration of IVIG can develop CAA; therefore, the difference between IVIG-responsive and IVIG-resistant patients and many scoring systems predicting the responsiveness to IVIG have been explored [12, 14, 15]. In addition, a few bioinformatic analyses have been introduced to uncover potential biomarkers of KD [16–19]. For our study, we aimed to discover potential biomarkers for the early diagnosis and treatment of KD. This study is the first to integrated WGCNA, LASSO regression and CytoHubba in Cytoscape to predict node genes of KD with the combination of two datasets from the public database. One independent dataset of KD pre- and post- IVIG therapy was gathered for further validation. In addition, the relationships between the infiltration of immune cells and node genes were analyzed to scale up our understanding of KD from the perspective of molecular immune mechanisms. Furthermore, the establishment of ceRNA networks of node genes would elucidate the mechanism of KD at the RNA regulatory level. In summary, this study that hybridized the aforementioned bioinformatic analysis might have the capability of providing candidate biomarkers for the early diagnosis and treatment of KD.

## Material and methods

### Microarray data

In Gene Expression Omnibus (GEO) (https://www.ncbi.nlm.nih.gov/geo/) database, four publicly available datasets related to Kawasaki disease (KD) were retrieved, and no further approval from the local ethics committee was required. GSE68004 [20] and GSE73461 [10] were both based on GPL10558 Illumina HumanHT-12 V4.0 expression beadchip platform. GSE68004 includes 76 KD patients and 37 health controls (HC), and GS73461 contains 77 KD patients and 55 HC (the information of one KD patient was missed). Because of the similar sample size and the same platform, these two datasets were incorporated to perform combined analysis, which was defined as the training set. GSE18606 [21] was assayed on GPL6480 Agilent-014850 Whole Human Genome Microarray, which was regarded as the validation set, and 20 KD patients and 9 HC were chosen. GSE63881 [12] was set as the validation set for KD patients following IVIG, based on the same platform as the training set and covering 171 acute (before IVIG) and 170 convalescent (after IVIG) KD patients. The detailed information of the four datasets was shown in Table 1.

**Table 1:** Characteristics of GEO datasets in this study

Tissue	Dateset ID	Country	No.of samples	GPL ID	No.of rows per platform	Usage here	References
Blood	GSE68004	USA	76KD, 37HC	GPL10558	48,107	combined analysis	Jaggi et al., 2018 [20]
Blood	GSE73461	UK	77KD, 55HC	GPL10558	48,107	combined analysis	Wright et al., 2018 [10]
Blood	GSE18606	USA	20KD, 9HC	GPL6480	41,108	validation set	Fury et al., 2010 [21]
Blood	GSE63881	Singapore	171A, 170C	GPL10558	48,107	Validation set (KD following IVIG)	Hoang et al., 2014 [12]

*A* Acute KD patients, *C* Convalescent KD patients

### Identification of differentially expressed genes (DEGs) by combined analysis

GSE68004 and GSE73461 were combined with the “*inSilicoMerging*” package [22]. Then batch normalization was performed through the *ComBat* method in the “*sva*” R package [23]. A platform annotation file was used to convert the probe expression into a gene expression matrix, and noncoding RNAs (ncRNAs) were removed from this matrix. DEGs between KD and HC were acquired by the eBayes function in the “limma” package [24]. A heatmap of the top ten upregulated and top ten downregulated genes was generated between KDs and HCs using the “*pheatmap*” package in R [25].

### Weighted gene coexpression network analysis (WGCNA)

We applied WGCNA to explore the interactions between genes and genes and between genes and clinical traits. The expression profile of the DEGs from the combined analysis was utilized to conduct WGCNA through the “*WGCNA package*” in R [7]. First, the *hclust* function was used to assess the presence of any obvious outliers and cluster the samples by a hierarchical clustering algorithm. Second, the optimal soft threshold for the adjacency matrix was selected with the *pickSoftThreshold* and *softConnectivity* functions so that the constructed correlation network was more approximate to the scale-free characteristic for the biological network. Third, *hierarchical clustering* and *dynamic tree cut* functions were implemented to identify gene modules that could sectionalize genes with the same expression pattern, and a minimum module size of 30 was adopted. The fourth step was to connect modules with the clinical traits, and in this study, we explored the relationships of the modules with disease and sex. Finally, gene significances (GS) and module memberships (MM) were obtained to recognize the hub genes with cutoffs of GS > 0.7 and MM > 0.9. GS was calculated to quantify the absolute value of associations between individual genes and the trait of interest (disease and sex in this analysis), while MM was the correlation of the module eigengene and gene expression profile. Module significance (MS), the average GS across genes in the module, was used to identify the hub module. Therefore, it is feasible to obtain hub genes in the hub module with GS and MM. Genes in the hub module were then retrieved to perform functional enrichment analysis with “*clusterProfiler*” R package [26] and the categories including biological process (BPs) in Gene Ontology (GO) and Kyoto Encyclopedia of Genes and Genomes (KEGG) pathways were selected [27]. *p*-value < 0.05 was considered to be criteria of significant enrichment. The top 10 BPs and KEGG pathways are displayed in the form of bubble plots with the aid of the “*ggplot2*” R package [28].

### Construction of a protein–protein interaction (PPI) network

The STRING database (http://string-db.org) [29] was used to identify known and predicted PPIs. Genes in the hub module were imported into STRING, and the interaction file was acquired and input into Cytoscape *(version 3.7.2)* for better visualization. The CytoHubba plugin of Cytoscape [30] was applied to score the top 5 genes by four algorithms incorporating MCC (maximal clique centrality), MNC (maximum neighborhood component), EPC (edge percolated component) degree and Betweenness. Genes that appeared in the top 5 genes of the five algorithms were retained.

**Fig. 1:**
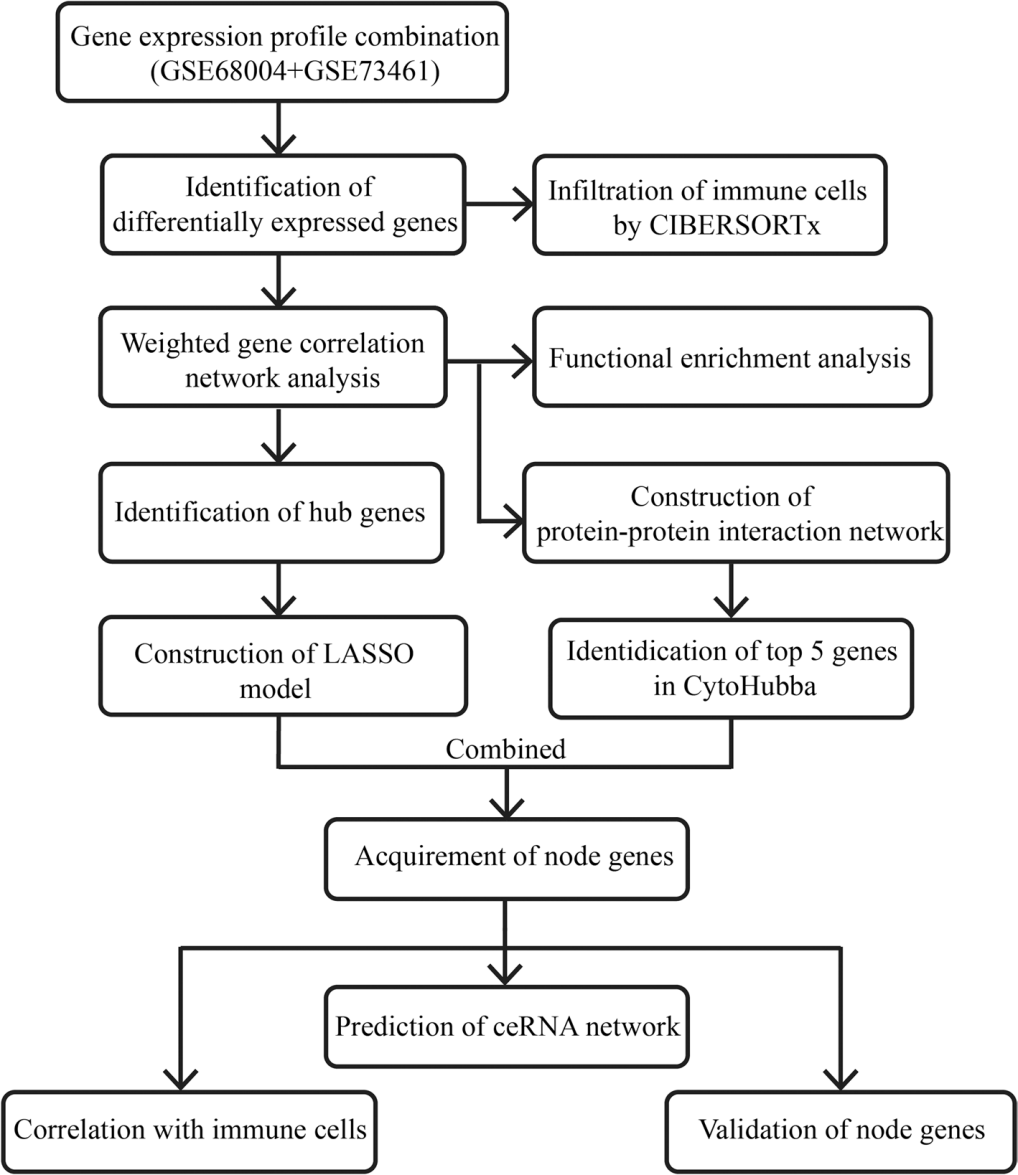
The workflow of the present study

### Construction of least absolute shrinkage and selection operator (LASSO) model

*LASSO* is a penalized regression method that uses the L1 penalty to restrain regression coefficients (λ) toward zero, and genes with nonzero coefficients are retained as seed genes. The expression profile of hub genes was transferred to the R platform to perform *LASSO* regression analysis, which is based on the “*glmnet*” package [9] with nfolds = 10. The best λ-value was selected according to the minimum criterion. The model index for each sample was constituted with the following equation:

$$\mathrm{index}=\mathrm{ExpGene}1\ast\mathrm{Coef}1+\mathrm{ExpGene}2\ast\mathrm{Coef}2+\mathrm{ExpGene}3\ast\mathrm{Coef}3+$$

The “*Coef*” indicates the regression coefficient of the gene that is rooted in the LASSO regression to weight the expression value of the selected gene, and “*Exp*” implies the expression value of the gene.

### Validation of the selected genes in datasets

ROC curves of all the selected genes in the training set and GSE18606, including genes retained in the LASSO model and the algorithms in the CytoHubba, were plotted with the roc function in the “pROC” package [31], and genes with area under the curve (AUC) > 0.9 in both sets were defined as node genes. Then batch effect of node genes between different platforms of the training set and validation set was eliminated using the “sva” R package. The difference in node genes was additionally investigated in GSE63881 to scrutinize the expression change following IVIG. GraphPad Prism (version 8.0.2) was used to construct violin plots of node genes in the three datasets, *p–*values between groups were calculated with the Wilcoxon test in GSE18606 and GSE63881. Principal component analysis (PCA) plots were used to display the efficacy of the node-gene signature to distinguish KDs from HCs, which were analyzed and visualized using the “limma” and “ggplot2” packages, respectively.

**Fig. 2:**
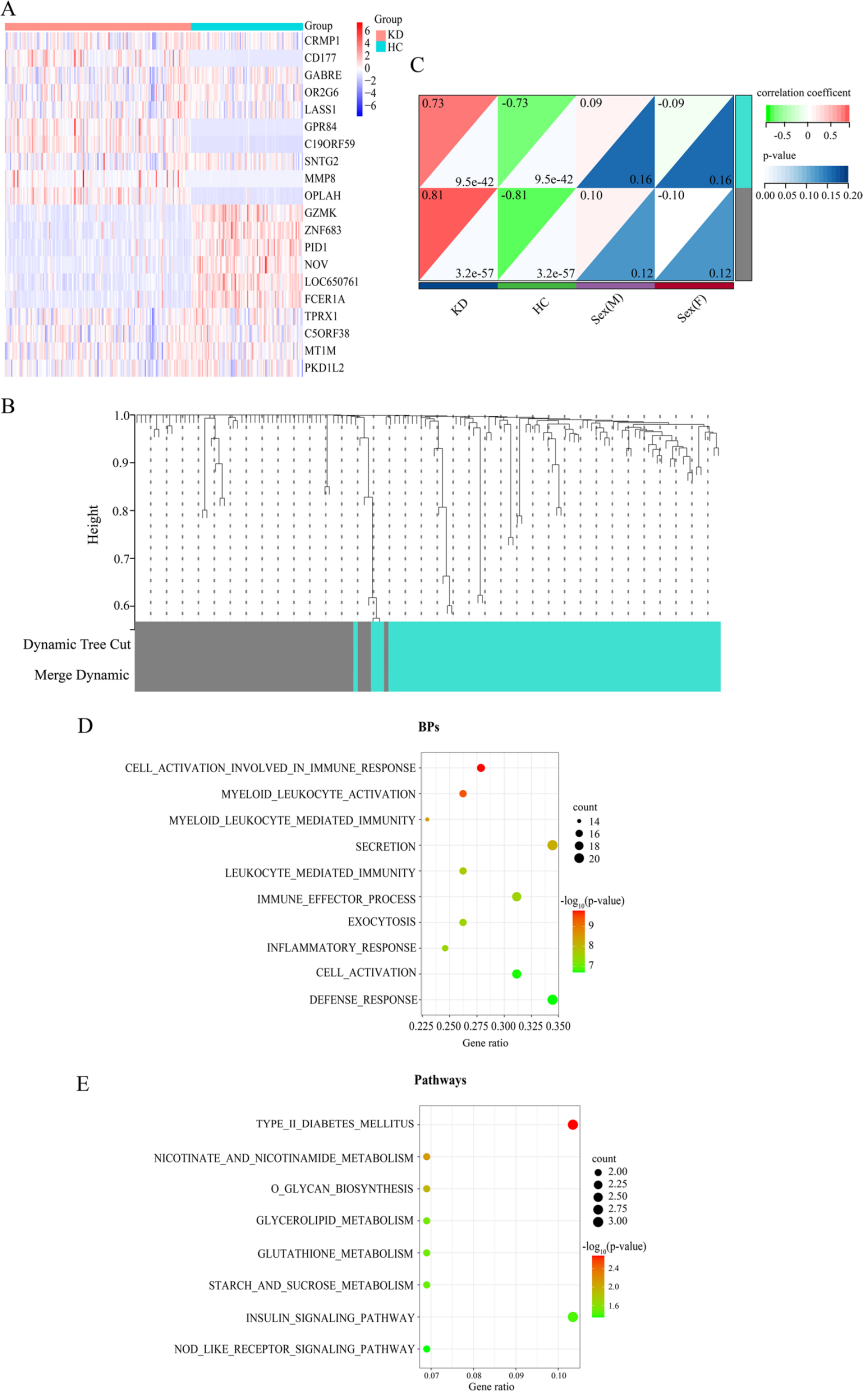
Weighted gene correlation network analysis (WGCNA) and functional annotations of the hub genes. **A** The heatmap of top 10 up- and down-regulated genes between Kawasaki disease (KD) patients and health controls (HC). Row: genes, Columns: samples. Colors indicate the gene expression level, in which red means high level and blue means low level. **B** Cluster dendrogram represents the distribution of genes with corresponding module colors, which incorporates a sum of 2 modules, and genes that don’t co-express with other genes are divided into the gray module. **C** Module-trait correlation heatmap. Numbers in the upper left corners represent the correlation coefficient of modules to traits, red color represents positive correlation, and green color represents negative correlation. Numbers in the lower right corners means the *p*-values. Each row symbolizes a module eigengene and each column symbolizes a trait. Sex(F): female; Sex(M): male. **D** The bubble plot displays the significant enriched biological processes (BPs) of 80 genes. **E** The bubble plot shows the significant enriched pathways of 80 genes. The color of the dots refers to the -log_10_ (*p*-value), and the size of the dots refers to the number of DEGs mapped to the indicted pathways, respectively. The significant biological processes and pathways are selected according to *p*-value < 0.05

**Fig. 3:**
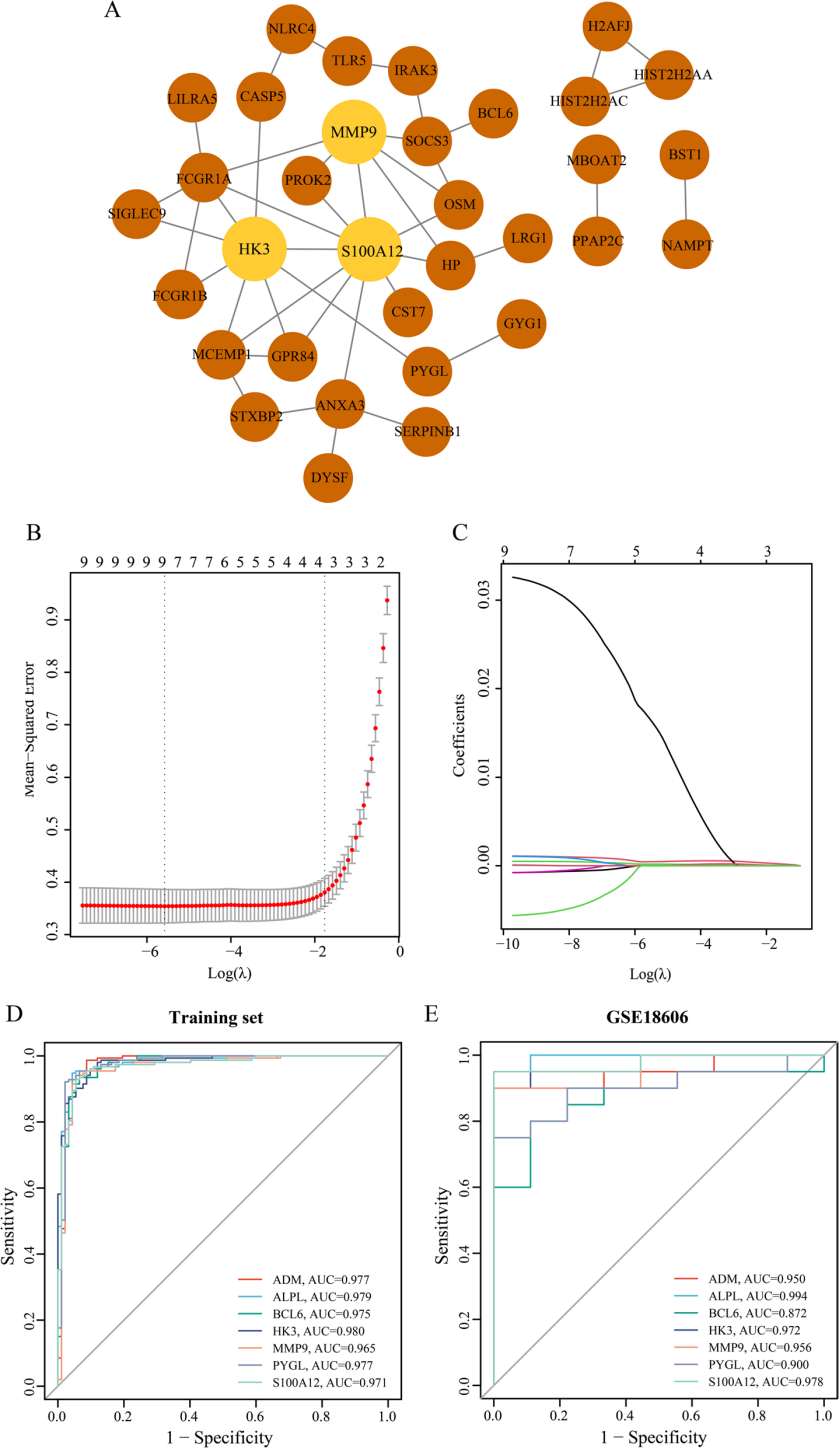
Screening of node genes. **A** PPI network map of 80 genes, which includes 33 nodes and 43 edges. Light brown circles stand for common genes appearing in the five algorithms. **B** *LASSO* model. λ = λ.1se, mean-squared error (MSE) = 0.02655. **C** The variation of coefficients of hub genes with different Log λ. ROC curves of the seven genes alone (ADM, ALPL, BCL6, HK3, MMP9, PYGL and S100A12) in both the training set (**D**) and validation set (GS18606) (**E**)

**Fig. 4:**
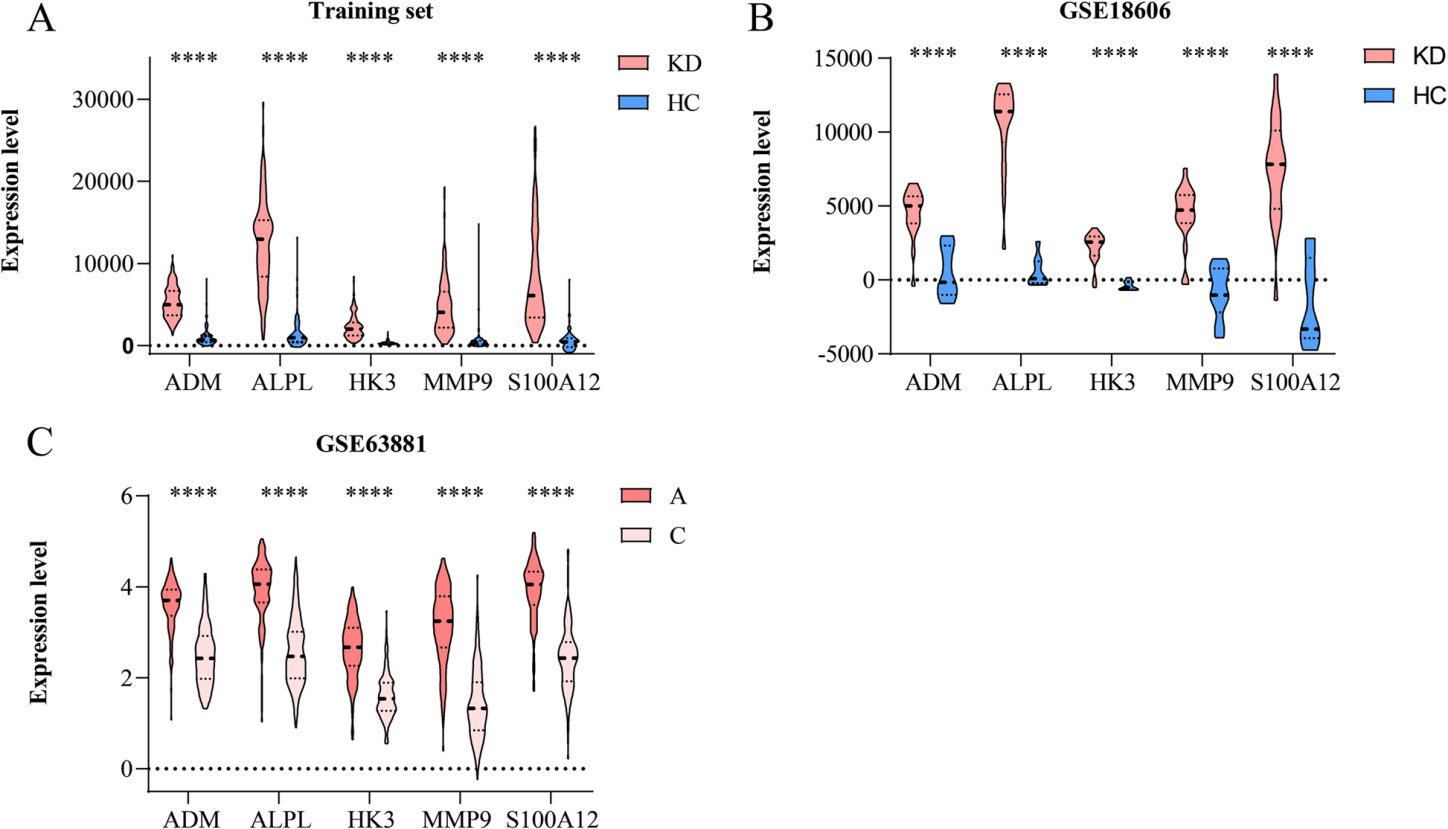
Validation of node genes. **A **Violin plots of node genes between KD and HC in training set. **B **Violin plots of node genes between KD and HC in GSE18606. **C **Violin plots of node genes between A and C in GSE63881. Pink represents KD, blue represents HC, deep pink refers to A, and light pink refers to C. (****P < 0.0001). **“**A” and “C” means acute and convalescent KD patients, separately

**Fig. 5:**
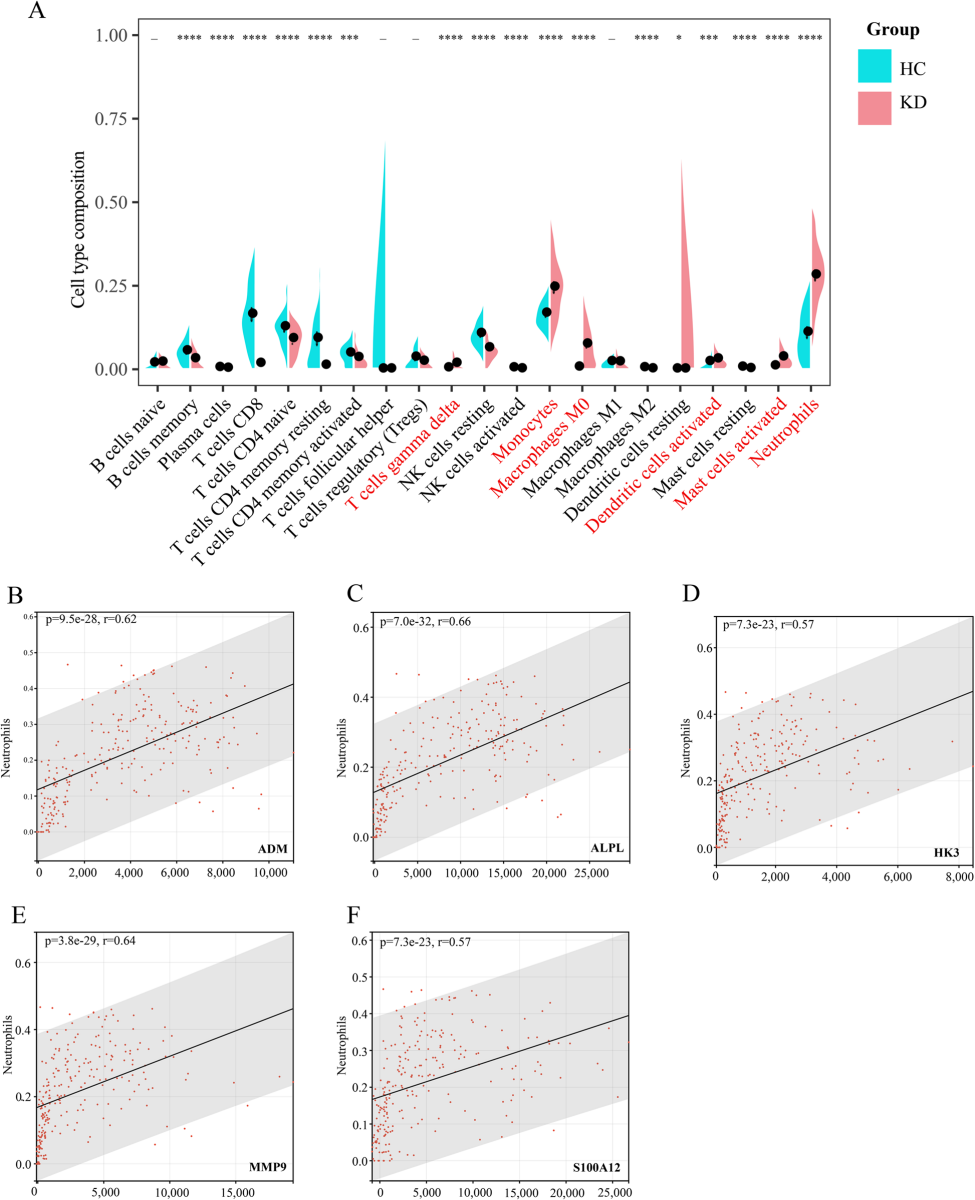
Immune cell infiltration and correlation with genes. **A** Comparison of 22 immune cell subtypes between KDs and HCs. *p*-values are obtained by the *Wilcoxon test*. Pink and turquoise color represent KDs and HCs, respectively (_: non-significant, *P < 0.05, **P < 0.01, ***P < 0.001, ****P < 0.0001) (The fraction of eosinophils is zero.). The immune cells displayed with red color are upregulated prominently in KD. Correlation between ADM and neutrophils (**B**); ALPL and neutrophils (**C**); HK3 and neutrophils (**D**); MMP9 and neutrophils (**E**); S100A12 and neutrophils (**F**). “r” means the correlation coefficient between nodes and immune cells

**Fig. 6:**
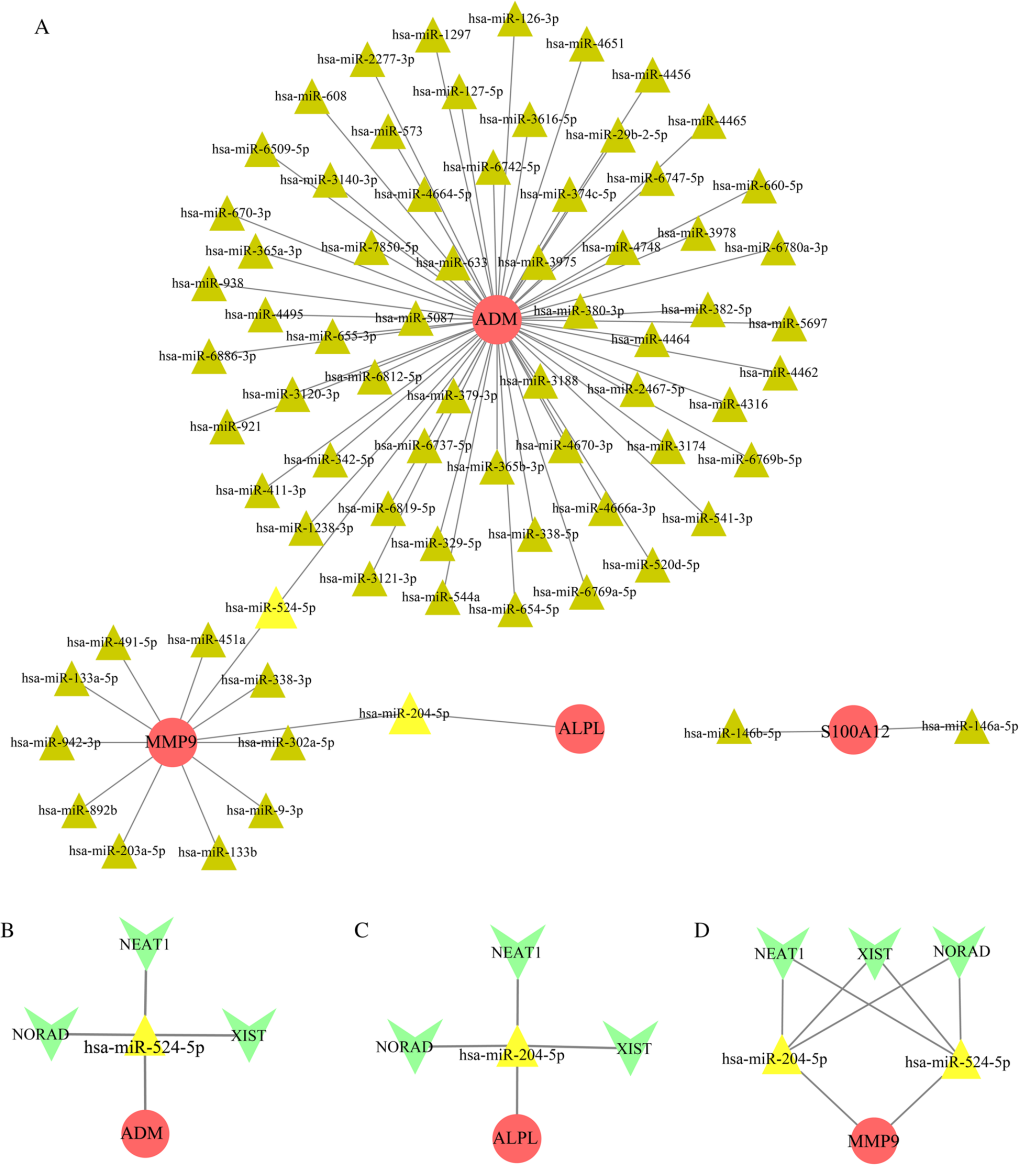
Prediction of ceRNA network. **A **The miRNA-mRNA interaction network includes 79 nodes and 77 edges. The ceRNA networks of NEAT1/NORAD/XIST-hsa-miR-524-5p-ADM (**B**), NEAT1/NORAD/XIST-hsa-miR-204-5p-ALPL (**C**), and NEAT1/NORAD/XIST-hsa-miR-524-5p/hsa-miR-204-5p-MMP9 (**D**). Red circles stand for node genes. Triangles stand for miRNAs (brown triangles: connect with only one mRNA; yellow triangles: connect with two mRNAs). And green Vs represent lncRNAs

### Immune cell infiltration analysis

To prove the difference in the relative proportions of infiltrating immune cells between KDs and HCs, a bioinformatic algorithm called CIBERSORTx (https://cibersortx.stanford.edu/) [32] was used. The abundance level of immune cells was assessed using the 22 kinds of immune cell subsets with 1000 permutations. GraphPad Prism (version 8.0.2) [33] and the Wilcoxon test were utilized to display and analyze the comparative expression of immune cells. A *p*-value < 0.05 was regarded as the criterion for a significant difference. The correlation analysis among immune cells and node genes was undertaken using the “corrplot” R package [34]. The relationships between node genes and the most relevant immune cells were determined by Spearman correlation analysis and are shown using the “ggplot2” package.

### Prediction of ncRNAs

MiRNAs targeting node genes were predicted by the miRTarBase database in the Enrichr platform (http://amp.pharm.mssm.edu/Enrichr/) [35] with the threshold of *p–*value < 0.05. The lncRNAs interacting with the remaining miRNAs were predicted through starBase (http://starbase.sysu.edu.cn/) [36], with the following criteria: mammalian, human h19 genome, CLIP-Data ≥ 5, and with or without degradome data. The coexpression networks of mRNAs-miRNAs and ceRNA networks based on mRNAs-miRNAs-lncRNAs were established and visualized utilizing Cytoscape (version 3.7.2).

### Statistical analysis

DEGs between KDs and HCs were defined by *p*-values < 0.05 and |log2 fold change (FC)|≥ 2. The Wilcoxon test was applied to compare the mRNA expression in the datasets and the proportions of immune cells between KDs and HCs, and *p*-values < 0.05 were considered to be significant. The cutoff value for the selection of BPs and KEGG pathways was *p*-value < 0.05. ROC curve analysis and PCA were performed to assess the diagnostic accuracy and value of the selected genes. The relationship between node genes and immune cells was calculated through Spearman’s rank correlation. And *p*-values < 0.05 were considered statistically significant for almost all results, and all tests were two-tailed. The visualization of the data was accomplished by R 4.0.5, GraphPad Prism (version 8.0.2) and Cytoscape (version 3.7.2).

## Results

### Identification of DEGs by combined analysis

Based on the workflow in Fig. 1, we concluded that after combined analysis of datasets GSE68004 and GSE73461, there were 267 DEGs with *p*-values < 0.05 and |log2 FC|≥ 2, in which 231 were upregulated and 36 were downregulated in KD. The top 10 upregulated and downregulated DEGs are presented in the heatmap (Fig. 2A).

### Modules associated with KD

The soft threshold power (β) of 28 was the optimal power, while the scale-free topology fit index was 0.86 along with a mild mean-connectivity (Figure S1), which made the network accord with the power-law distribution in WGCNA. Then, a total of two modules were finally obtained (Fig. 2B-C), of which the turquoise module exhibited the highest biological association with KD (correlation coefficient = 0.73, *P =* 9.5E-42; Fig. 2C), and we declared the turquoise module to be the hub module. No significant linkage was found between the module and the sex of the samples. The hub module contained a total of 80 DEGs, and nine genes upregulated in KD were selected as centrally located intramodular hub genes (ACSL1, ADM, ALPL, B4GALT5, BCL6, DYSF, LOC440731, PYGL, TLR5) with the cutoff criteria of GS > 0.7 and MM > 0.9. In addition, genes that could not be co-expressed with other genes were assigned to the grey module, which was ignored in our study. According to the enrichment analysis of the hub module, the biological processes (Fig. 2D) were mainly enriched in cell activation involved in immune response, myeloid leukocyte activation, myeloid leukocyte mediated immunity, secretion and leukocyte mediated immunity; the prominent pathways (Fig. 3E) included type II diabetes mellitus, nicotinate and nicotinamide metabolism, O-glycan biosynthesis, glycerolipid and glutathione metabolism.

### Filtration and validation of node genes

For the sake of screening node genes that contribute to KD, we not only constructed the PPI network of genes in the hub module, but also identified the seed genes among the hub genes through LASSO regression analysis. The PPI network of genes acquired from WGCNA was established, with 33 nodes and 43 edges (Fig. 3A). Then the top 5 genes identified by the five algorithms in CytoHubba were crossed to obtain the common genes (Table 2), with the results of S100A12, HK3 and MMP9. Besides, the LASSO model was constructed (Fig. 3B-C) with four genes (ADM, ALPL, BCL6 and PYGL) possessing non-zero coefficients when λ.1se = 0.17130. Detailed information of the results is displayed in Table 3. To confirm the efficiency of both the algorithms in CytoHubba and the LASSO model, ROC curve analysis was performed (training set: the AUC of ADM = 0.977, the AUC of ALPL = 0.979, the AUC of BCL6 = 0.975, and the AUC of HK3 = 0.980, the AUC of MMP9 = 0.965, the AUC of PYGL = 0.977, the AUC of S100A12 = 0.971, Fig. 3D; GSE18606: the AUC of ADM = 0.950, the AUC of ALPL = 0.994, the AUC of BCL6 = 0.872, and the AUC of HK3 = 0.972, the AUC of MMP9 = 0.956, the AUC of PYGL = 0.900, the AUC of S100A12 = 0.978, Fig. 3E). The node genes were acquired according to their AUC values in two datasets, which incorporated ADM, ALPL, HK3, MMP9 and S100A12. For validation, violin plots which presented clearly separated trend (Fig. 4A-B) in the two datasets, and PCA (Figure S2A-B) suggesting a striking difference between KDs and HCs were conducted. In short, all of these genes effectively distinguish KDs from HCs, which also indicated the prominent predictive precision of both the LASSO model and algorithms in CytoHubba. In addition, these genes were markedly downregulated following IVIG treatment (Fig. 4C), which demonstrated that node genes were related to immune and inflammatory responses in KD and might be potential therapeutic targets for IVIG treatment.

**Table 2:** The scores of the top 5 genes from MCC, MNC, EPC, Degree and Betweenness algorithms of CytoHubba

Algorithms	Rank	Name	Score
Betweeness	1	S100A12	300.000
	2	HK3	210.333
	3	MMP9	108.667
	4	SOCS3	106.833
	5	ANXA3	105.333
Degree	1	S100A12	10.000
	2	HK3	8.000
	3	FCGR1A	6.000
	3	MMP9	6.000
	5	ANXA3	4.000
EPC	1	S100A12	13.646
	2	HK3	13.159
	3	FCGR1A	12.812
	4	MMP9	12.767
	5	MCEMP1	11.988
MCC	1	S100A12	18.000
	2	HK3	14.000
	3	MMP9	10.000
	4	FCGR1A	9.000
	5	MCEMP1	7.000
MNC	1	S100A12	8.000
	2	MMP9	6.000
	2	HK3	6.000
	4	FCGR1A	5.000
	5	OSM	3.000

**Table 3:** The specific information of the selected hub genes between KDs and HCs in the training set. Coefficient is the result of genes obtained from LASSO regression analysis

Gene	logFC	*P*–Value	Coefficience
ACSL1	2.648	1.19E-46	-
ADM	2.846	6.47E-64	0.000125746
ALPL	3.507	1.44E-62	2.68362E-05
B4GALT5	2.048	1.22E-63	-
BCL6	2.437	1.22E-62	1.96294E-08
DYSF	2.629	2.58E-54	-
LOC440731	2.687	4.46E-69	-
PYGL	2.382	1.01E-65	4.23351E-05
TLR5	3.095	1.54E-59	-

### Immune cell infiltration and correlation analysis with node genes

To obtain a deeper understanding of the mechanisms of KD, we explored and compared the composition of the infiltrating immune cells between KDs and HCs in the training set (Fig. 5A), eosinophils had an estimated abundance of 0). The proportions of gamma delta T cells (*P <* 0.000001), monocytes (*P <* 0.000001), M0 macrophages (*P <* 0.000001), activated dendritic cells (*P =* 0.00082), activated mast cells (*P <* 0.000001) and neutrophils (*P <* 0.000001) were higher in KDs. However, the fractions of memory B cells (*P <* 0.000001), plasma cells (*P =* 0.00082), CD8+ T cells (*P <* 0.000001), CD4+ naïve T cells (*P =* 0.000011), CD4+ resting memory T cells (*P <* 0.000001), CD4+ activated memory T cells (*P =* 0.00027), resting NK cells (*P <* 0.000001), activated NK cells (*P <* 0.000001), M2 macrophages (*P <* 0.000001), resting dendritic cells (*P =* 0.03) and resting mast cells (*P <* 0.000001) were higher in HCs. We then computed the correlation among the five node genes (ADM, ALPL, HK3, MMP9 and S100A12) and immune cells, as shown in Figure S3. The correlation between node genes and neutrophils, that were most positively associated with node genes, were illustrated as follows: ADM (*r* = 0.62, *P <* 0.0001, Fig. 5B), ALPL (*r* = 0.66, *P <* 0.0001, Fig. 5C), HK3 (*r* = 0.64, *P <* 0.0001, Fig. 5D), MMP9 (*r* = 0.64, *P <* 0.0001, Fig. 5E) and S100A12 (*r* = 0.57, *P <* 0.0001, Fig. 5F).

### Prediction of ncRNAs and construction of ceRNA networks

MiRNAs can induce gene degradation by binding to the 3’UTR of target mRNAs, and lncRNAs can competitively bind miRNAs, which are known as competing endogenous RNAs (ceRNAs). Both miRNAs and lncRNAs have been claimed to be involved in KD [44, 46, 47]; hence, we analyzed the target lncRNAs of the miRNAs interacting with node genes through online databases. The mRNA‒miRNA pairs were presented in Fig. 6A and Table 4, with 79 nodes and 77 edges. The top 2 most significant miRNAs were hsa-miR-524-5p (*p*-value = 0.000723) and hsa-miR-204-5p (*p*-value = 0.003814884), and they connected with two node genes. On the basis of starBase, the co-associative lncRNAs are NEAT1, NORAD and XIST. A total of three ceRNA networks were ultimately constructed: NEAT1/NORAD/XIST-hsa-miR-524-5p-ADM (Fig. 6B), NEAT1/NORAD/XIST-hsa-miR-204-5p-ALPL (Fig. 6C), NEAT1/NORAD/XIST-hsa-miR-524-5p/hsa-miR-204-5p-MMP9 (Fig. 6D).

**Table 4:** The miRNA-mRNA networks

mRNA	miRNAs
ADM	hsa-miR-524-5p,hsa-miR-4462,hsa-miR-660-5p,hsa-miR-4456,hsa-miR-126-3p,hsa-miR-2277-3p,hsa-miR-6509-5p,hsa-miR-938,hsa-miR-6886-3p,hsa-miR-921, hsa-miR-411-3p,hsa-miR-380-3p,hsa-miR-3975,hsa-miR-633,hsa-miR-5087,hsa-miR-379-3p,hsa-miR-3188,hsa-miR-365b-3p,hsa-miR-4670-3p,hsa-miR-2467-5p, hsa-miR-4464,hsa-miR-4748,hsa-miR-374c-5p,hsa-miR-6742-5p,hsa-miR-4664-5p,hsa-miR-7850-5p,hsa-miR-655-3p,hsa-miR-6812-5p,hsa-miR-6737-5p, hsa-miR-6819-5p,hsa-miR-329-5p,hsa-miR-338-5p,hsa-miR-4666a-3p,hsa-miR-3174,hsa-miR-4316,hsa-miR-382-5p,hsa-miR-3978,hsa-miR-6747-5p, hsa-miR-29b-2-5p,hsa-miR-3616-5p,hsa-miR-127-5p,hsa-miR-573,hsa-miR-3140-3p,hsa-miR-365a-3p,hsa-miR-4495,hsa-miR-3120-3p,hsa-miR-342-5p, hsa-miR-1238-3p,hsa-miR-3121-3p,hsa-miR-544a,hsa-miR-654-5p,hsa-miR-6769a-5p,hsa-miR-520d-5p,hsa-miR-541-3p,hsa-miR-6769b-5p,hsa-miR-5697, hsa-miR-6780a-3p,hsa-miR-4465,hsa-miR-4651,hsa-miR-1297,hsa-miR-608,hsa-miR-670-3p
ALPL	hsa-miR-204-5p
MMP9	hsa-miR-524-5p,hsa-miR-204-5p,hsa-miR-451a,hsa-miR-133a-5p,hsa-miR-942-3p,hsa-miR-892b,hsa-miR-203a-5p,hsa-miR-133b,hsa-miR-9-3p,hsa-miR-302a-5p, hsa-miR-338-3p,hsa-miR-491-5p
S100A12	hsa-miR-146b-5p,hsa-miR-146a-5p

## Discussion

KD is a self-limited multisystemic vasculitis that develops in genetically susceptible children after exposure to stimuli, mostly microbial and viral infections. Marked activation of immune and inflammatory reactions plays important roles in the development of KD [37, 38]. IVIG, which is a plasma-derived polyclonal IgG preparation, is considered the first-line therapy for KD and can strikingly decrease the incidence of CAA. However, without specific biomarkers, it is difficult to diagnose and give timely treatment at the early stage, which elevates the probability of CAA. Accordingly, the purpose of this research was to identify potential diagnostic biomarkers and possible mechanisms of KD through bioinformatic analysis and to offer an original direction for recognizing and treating KD patients.

We mainly combined two bioinformatic analyses to identify the underlying biomarkers of KD. First, WGCNA is a system biology approach which can group genes into modules with the same expression patterns, and correlate clinical features with modules. Compared with concentrating on DEGs, WGCNA can use the information of thousands of genes with the greatest variations to identify the interesting gene sets [39]. Many kinds of cancer such as breast [40] and gastric cancer [41], and other diseases like KD [18, 42] have been analyzed by WGCNA. And in this study, the turquoise module was identified as the hub module according to the results of WGCNA, and the included genes were subjected to *clusterprofiler* R package. The dominant enriched BP terms in the GO analysis were cell activation involved in immune response, myeloid leukocyte activation, myeloid leukocyte mediated immunity, secretion and leukocyte mediated immunity biological processes [48, 49]; the prominent pathways included type II diabetes mellitus, nicotinate and nicotinamide metabolism, O-glycan biosynthesis, glycerolipid and glutathione metabolism.

Second, LASSO regression analysis is a complex algorithm, that uses regularization to enhance the accuracy of prediction. And it was applied to the expression matrix of candidate genes to construct the prognostic multi-gene signature. Multiple RNA risk signatures were identified through LASSO penalized Cox regression analysis [43, 44]. Besides, the combination of these two methods has become a tendency recently, characteristic genes were determined in endometriosis [45] and sepsis [46, 47] with the application of the combination of WGCNA and LASSO. Regarding the gene signature of our analysis, a five-gene signature was formed after combining the top five genes of the five algorithms in CytoHubba and the retained genes in LASSO analysis, comprising ADM, ALPL, HK3, MMP9 and S100A12, all were significantly upregulated in KD and with good validation performance in other cohorts.

Adrenomedullin (ADM) is a vasoactive peptide mediating vasodilation and endothelial function regulation. The expression level of ADM has been reported to be elevated in KD [48, 49], and higher in KD patients developed CAAs [48]. Plasma ADM levels are positively correlated to the degree of endothelial damage in atherosclerosis patients [50], which indicate that it is a good indicator of the prognosis in patients with CA disease [51]. Alkaline phosphatase (ALPL), which could regulate tissue mineralization and execute an integral function in cardiovascular remodeling [52], has been identified to be increased in acute KD patients and decreased after IVIG infusion [53], which are concordant with our analysis. In addition, ALPL could predict the morbidity and mortality of cardiovascular-related disease [54, 55], and is associated with inflammation [56]. Hexokinase 3 (HK3) is involved in the glucose metabolic pathway and also correlated with immune cell infiltration [57]. HK3 has been detected to be upregulated in IVIG-non responders of KD [58], and further examination should be conducted in both IVIG-non responders and responders of acute KD. Matrix metalloproteinase-9 (MMP9), a member of MMPs family, could degrade extracellular matrix components and assume an important role in the processes of inflammation and tissue remodeling. Elevated expression of MMP9 has been reported in acute KD [59] through mediating vascular smooth muscle cell (VSMC) migration and neointimal formation, besides, it is also related to the formation of CAA in KD [60–62]. S100 calcium-binding protein A12 (S100A12) belongs to the S100 protein family and regulates amounts of inflammatory responses. S100A12 is significantly upregulated in acute KD and declined after IVIG treatment [53, 63–65], in accordance with aforementioned results. While in patients with giant CAAs, plasma S100A12 levels remain elevated [53, 63]. Besides, S100A12 could promote vasculitis by stimulating the production of IL-1β, which directly induces CA endothelial cell dysfunction [64] and impacts the risk of the formation of CAL in children with KD [65].

The infiltration profile of 22 immune cells in KD and HC was assessed using CIBERSORTx. Gamma delta T cells, monocytes, M0 macrophages, activated dendritic cells, activated mast cells and neutrophils were significantly augmented in KDs, which may explain the contribution of innate immunity to the occurrence of vasculitis in KD [66–68]. The fractions of memory B cells, plasma cells, CD8^+^ T cells, CD4^+^ naïve T cells, CD4^+^ resting memory T cells, CD4^+^ activated memory T cells, resting NK cells, activated NK cells, M2 macrophages, resting dendritic cells and resting mast cells were decreased in KDs, which has been demonstrated in several studies [13, 69–72]. Furthermore, by performing correlation analysis between node genes and immune cells, we discovered that all the node genes have correlation with neutrophils, which has been demonstrated in previous studies [73–77]. Therefore, we can infer that the five-gene signature is involved in the process of immune cell-mediated occurrence of KD, and our study provides a reference for the study of immune mechanisms in the pathogenesis of KD.

Target miRNAs and the target lncRNAs of these miRNAs were predicted for the node genes, which may reveal the mechanism through which the node genes are adjusted at the transcriptome level. Hsa-miR-524-5p and hsa-miR-204-5p have been found to be considerably downregulated in patients with heart disease as compared to controls [78, 79]. LncRNA-NEAT1 is associated with NLRP3 inflammasome to augment their aggregation and can also promote the production of IL-1β [80, 81]. Besides, it could regulate the expression of chemokines and cytokines, such as IL-6 [82]. NORAD modulates inflammation and atherosclerosis in various cardiovascular diseases [83]. Furthermore, XIST is involved in the pathogenesis of SLE and rheumatoid arthritis (RA) [84] and can promote the expression of MMP3 and caspase-3 [85], which are important molecules contributing to KD [86, 87]. Finally, three intact ceRNA network(s) was constructed, namely, NEAT1/NORAD/XIST-hsa-miR-524-5p-ADM, NEAT1/NORAD/XIST-hsa-miR-204-5p-ALPL, NEAT1/NORAD/XIST-hsa-miR-524-5p/hsa-miR-204-5p-MMP9. We hypothesize that this ceRNA network has a pivotal role in KD.

However, the disadvantage of our study is obvious. Compared to RNA-seq technique, which sequences the entire transcriptome, the datasets employed in our study only provide the profiles of predefined transcripts or genes through hybridization which could not reveal the full picture of gene expression. Besides, our analysis is based on prediction, and the exact function of each molecule in the process of KD needs to be verified by further cell and animal experiments. In addition, a larger cohort is needed to investigate the unknown process underlying KD. And the clinical information of samples was not comprehensive, which may prevent us from discovering other influence factors.

## Conclusion

In conclusion, we identified a five-gene signature with the aid of WGCNA, LASSO regression analysis and algorithms in Cytoscape, and ultimately acquired three integrated ceRNA network: NEAT1/NORAD/XIST-hsa-miR-524-5p-ADM, NEAT1/NORAD/XIST-hsa-miR-204-5p-ALPL, NEAT1/NORAD/XIST-hsa-miR-524-5p/hsa-miR-204-5p-MMP9, with online databases. Our bioinformatic analysis offers potential biomarkers for the early diagnosis of KD and might perfect the regulatory mechanism from the perspective of ceRNA networks.

## Supplementary Information

**Additional file 1: Figure S1.** Soft threshold analysis is used to acquire the scale-free fit index of network topology.

**Additional file 2: Figure S2. **Validation of hub genes. Principal component analyses (PCA) of the training set** (A)** and GSE18606 **(B)**. Red circles represent KD, turquoise circles represent HC.

**Additional file 3: Figure S3. **Correlation among five node genes and immune cells in the training set. The number and the color in the dots mean the strength of the correlation; red indicates the positive correlation, and blue represents the negative. And the asterisk in the dots means the *p*-value (-: non-significant, *P<0.05, **P<0.01, ***P<0.001, ****P<0.0001).

**Additional file 4: Table S1.** The results of all the statistical tests. DEGs are the genes with *p*-values < 0.05 and |log_2_ FC| ≥ 2 between KD and HC. GENES_IN_MODULES presents the modular distribution of genes according to the clustering of the WGCNA analysis. ENRICHED_GO_OF_HUB_MODULE and ENRICHED_PATHWAYS_OF_HUB_MODULE are the results of the enrichment analysis of genes in hub module using “*clusterprofiler*” R package. CIBERSORTx presents the proportions of immune cells in KD and HC, and the *p*-values between them.

## Data Availability

The following data were offered considering data availability: The original microarray data is available at Gene Expression Omnibus: GSE68004 (https://www.ncbi.nlm.nih.gov/geo/query/acc.cgi?acc=GSE68004), GSE73461 (https://www.ncbi.nlm.nih.gov/geo/query/acc.cgi?acc=GSE73461), GSE18606 (https://www.ncbi.nlm.nih.gov/geo/query/acc.cgi?acc=GSE18606), GSE63881 (https://www.ncbi.nlm.nih.gov/geo/query/acc.cgi?acc=GSE63881). All data obtained in this paper are contained in the figures and tables (Figs. 1, 2, 3, 4, 5 and 6; Tables 1, 2, 3 and 4; Figures S1, S2 and S3; Table S1).

## Abbreviations

A: Acute KD patients
ADM: Adrenomedullin
ALPL: Alkaline phosphatase
AUC: Area under the curve
BP: Biological process
C: Convalescent KD patients
CA: Coronary arteries
CAA: Coronary artery aneurysm
CAL: Coronary artery lesion
ceRNAs: Competing endogenous RNAs
CRP: C-reactive protein
DEGs: Differential expressed genes
EPC: Edge Percolated Component
FC: Fold change
FDR: False discovery rate
GEO: Gene Expression Omnibus
GO: Gene Ontology
GS: Gene significances
HC: Health controls
HK3: Hexokinase 3
Ig: Immunoglobulin
IL-6: Interleukin-6
IVIG: Intravenous immunoglobulin
KD: Kawasaki disease
KEGG: Kyoto Encyclopedia of Genes and Genomes
LASSO: Least absolute shrinkage and selection operator
MCC: Maximal Clique Centrality
MM: Module memberships
MMP-9: Matrix metalloproteinase 9
MNC: Maximum Neighborhood Component
MS: Module significance
ncRNAs: Noncoding RNAs
PCA: Principal component analysis
PPI: Protein–protein interaction
RA: Rheumatoid arthritis
ROC: Receiver operating characteristic
S100A12: S100 calcium-binding protein A12
SLE: Systemic lupus erythematosus
VSMC: Vascular smooth muscle cell
WGCNA: Weighted gene correlation network analysis
