# Supplementary figures and images for: Bioinformatic analysis of underlying mechanisms of Kawasaki disease via Weighted Gene Correlation Network Analysis (WGCNA) and the Least Absolute Shrinkage and Selection Operator method (LASSO) regression model
Source: BMC Pediatr. 2023 Feb 24;23:90. doi: 10.1186/s12887-023-03896-4 (PMC9951419; doi:10.1186/s12887-023-03896-4)

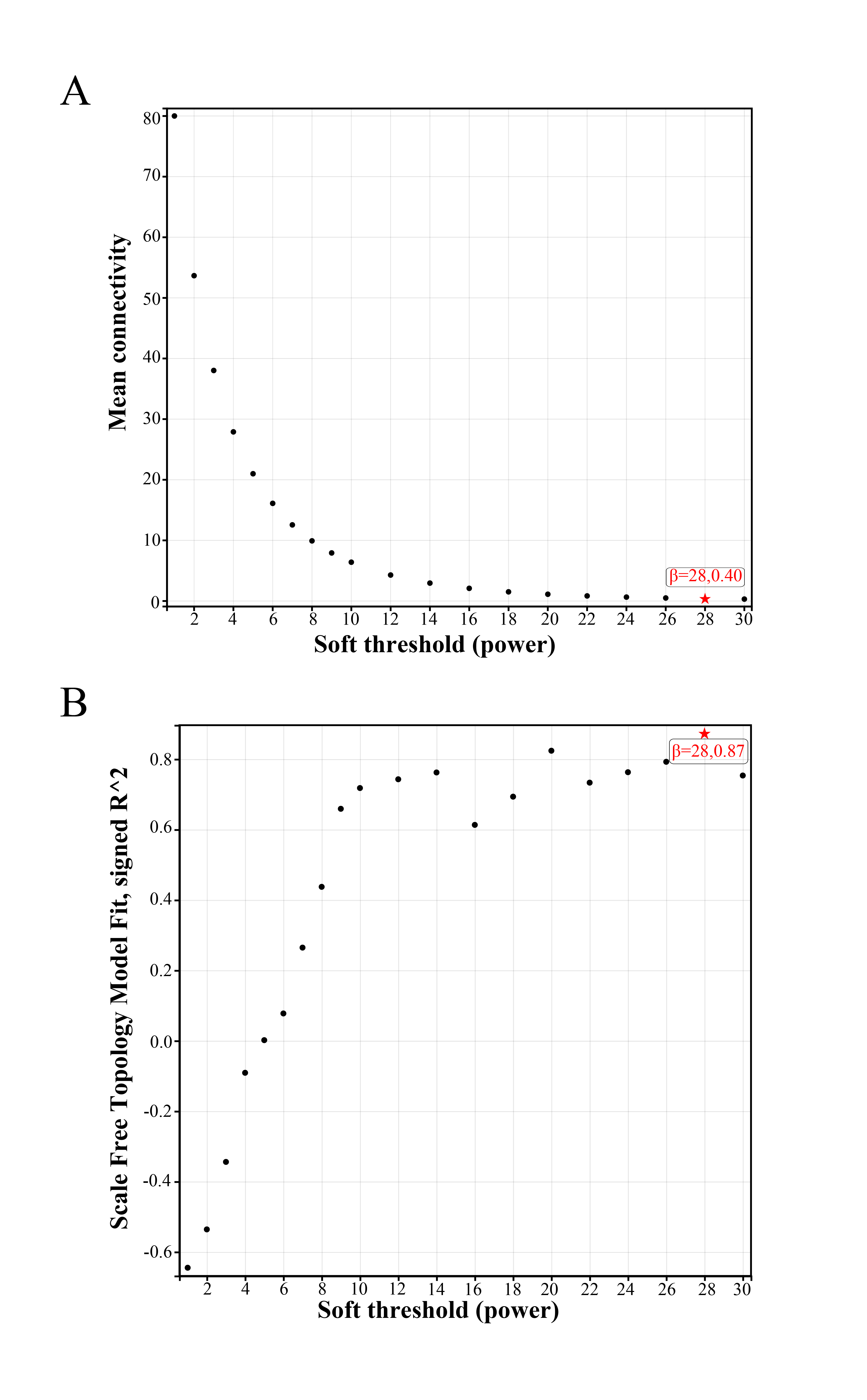

Supplement: Supplementary file 1 — Additional file 1: Figure S1. Soft threshold analysis is used to acquire the scale-free fit index of network topology. [file 12887_2023_3896_MOESM1_ESM.tif]

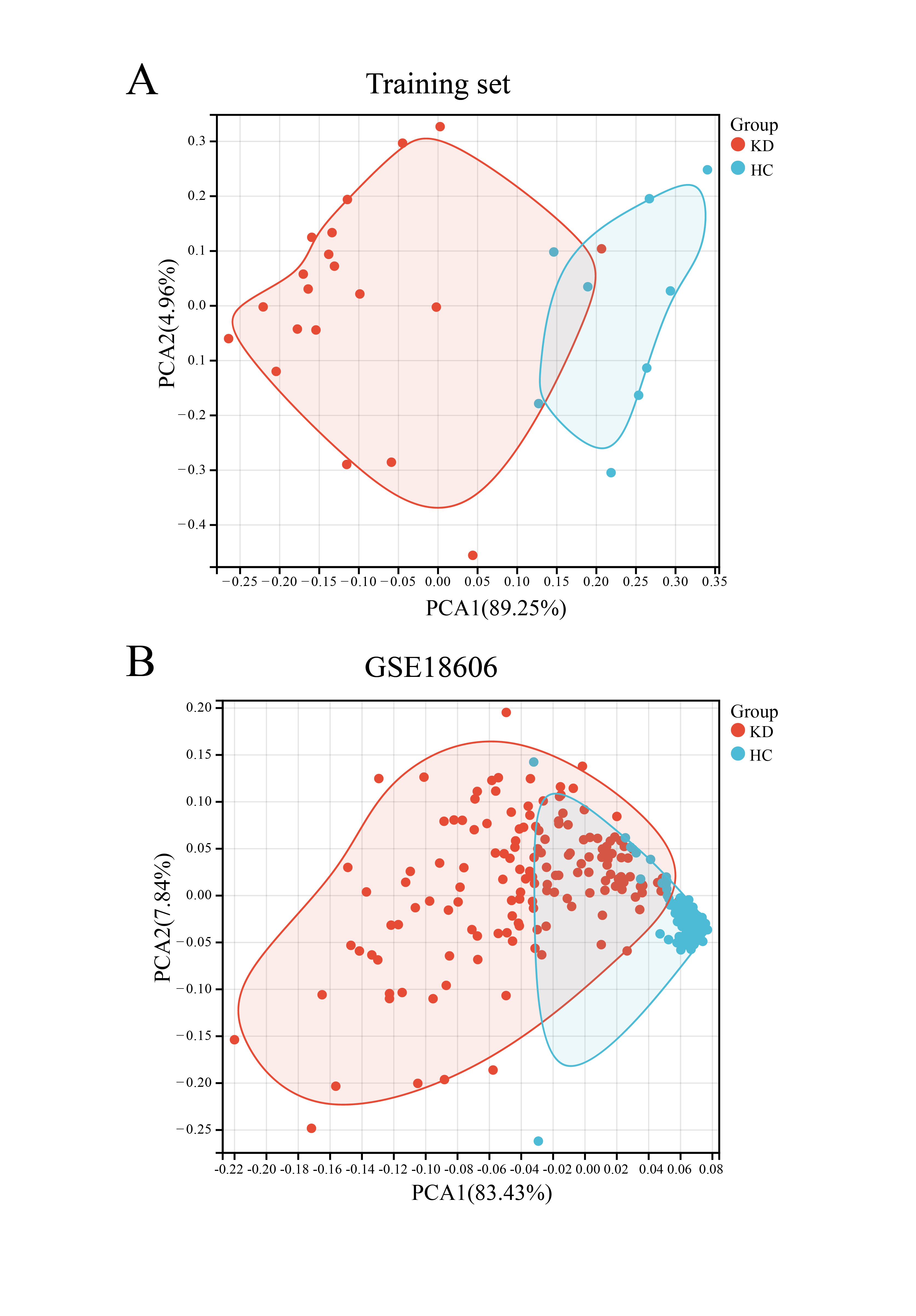

Supplement: Supplementary file 2 — Additional file 2: Figure S2. Validation of hub genes. Principal component analyses (PCA) of the training set (A) and GSE18606 (B). Red circles represent KD, turquoise circles represent HC. [file 12887_2023_3896_MOESM2_ESM.tif]
